# Comparison of the systemic inflammatory response syndrome between monomicrobial and polymicrobial *Pseudomonas aeruginosa *nosocomial bloodstream infections

**DOI:** 10.1186/1471-2334-5-94

**Published:** 2005-10-31

**Authors:** Alexandre R Marra, Gonzalo ML Bearman, Richard P Wenzel, Michael B Edmond

**Affiliations:** 1 Department of Infectious Diseases, Universidade Federal de São Paulo, São Paulo, Brazil; 2 Department of Internal Medicine, Medical College of Virginia Campus, Virginia Commonwealth University, Richmond, Virginia, USA

## Abstract

**Background:**

Some studies of nosocomial bloodstream infection (nBSI) have demonstrated a higher mortality for polymicrobial bacteremia when compared to monomicrobial nBSI. The purpose of this study was to compare differences in systemic inflammatory response and mortality between monomicrobial and polymicrobial nBSI with *Pseudomonas aeruginosa*.

**Methods:**

We performed a historical cohort study on 98 adults with *P. aeruginosa *(Pa) nBSI. SIRS scores were determined 2 days prior to the first positive blood culture through 14 days afterwards. Monomicrobial (n = 77) and polymicrobial BSIs (n = 21) were compared.

**Results:**

78.6% of BSIs were caused by monomicrobial *P. aeruginosa *infection (MPa) and 21.4% by polymicrobial *P. aeruginosa *infection (PPa). Median APACHE II score on the day of BSI was 22 for MPa and 23 for PPa BSIs. Septic shock occurred in 33.3% of PPa and in 39.0% of MPa (p = 0.64). Progression to septic shock was associated with death more frequently in PPa (OR 38.5, CI95 2.9–508.5) than MPa (OR 4.5, CI95 1.7–12.1). Maximal SIR (severe sepsis, septic shock or death) was seen on day 0 for PPa BSI vs. day 1 for MPa. No significant difference was noted in the incidence of organ failure, 7-day or overall mortality between the two groups. Univariate analysis revealed that APACHE II score ≥20 at BSI onset, Charlson weighted comorbidity index ≥3, burn injury and respiratory, cardiovascular, renal and hematologic failure were associated with death, while age, malignant disease, diabetes mellitus, hepatic failure, gastrointestinal complications, inappropriate antimicrobial therapy, infection with imipenem resistant *P. aeruginosa *and polymicrobial nBSI were not. Multivariate analysis revealed that hematologic failure (p < 0.001) and APACHE II score ≥20 at BSI onset (p = 0.005) independently predicted death.

**Conclusion:**

In this historical cohort study of nBSI with *P. aeruginosa*, the incidence of septic shock and organ failure was high in both groups. Additionally, patients with PPa BSI were not more acutely ill, as judged by APACHE II score prior to blood culture positivity than those with MPa BSI. Using multivariable logistic regression analysis, the development of hematologic failure and APACHE II score ≥20 at BSI onset were independent predictors of death; however, PPa BSI was not.

## Background

*P. aeruginosa *is an important nosocomial BSI pathogen with a high associated mortality [1]. Although the frequency of Gram-negative sepsis has diminished over the last 20 years, the incidence of polymicrobial nBSI infection has increased [2]. In addition, the mortality associated with nosocomial bloodstream infections, particularly those occurring in the intensive care setting, is greater than that of community acquired BSI [3].

Prior studies of nosocomial bloodstream infection (nBSI) have reported a higher associated mortality with polymicrobial nBSI than with monomicrobial nBSI [4]. These studies compared polymicrobial infections with monomicrobial infections caused by diverse pathogens [5]. As such, few investigators have analyzed the clinical significance of polymicrobial versus monomicrobial BSI with a specific pathogen [6]. In addition, a major difficulty in interpreting prior studies is both the marked heterogeneity of variables and the varied definitions of polymicrobial infection [4,6,7]. Little information exists about the systemic inflammatory response in polymicrobial BSI [4,5].

The purpose of this study was to evaluate and compare the inflammatory response, clinical course, and outcomes of monomicrobial and polymicrobial nosocomial BSI due to *Pseudomonas aeruginosa*.

## Methods

### Setting

The Virginia Commonwealth University Medical Center (VCUMC) is an 820-bed tertiary care facility in Richmond, Virginia. The hospital houses 9 intensive care units (ICUs), including pediatric ICUs and a burn unit. Approximately 30,000 patients are admitted annually.

### Study design

Using the Surveillance and Control of Pathogens of Epidemiological Importance (SCOPE) database of bloodstream infections occurring at 49 U.S. hospitals [1], we identified all patients with a diagnosis of nBSI due to *P. aeruginosa *at VCUMC from January 1, 1996 through December 31, 2003. Patients were considered to have had BSI due to *P. aeruginosa *if ≥ 1 blood culture was positive for this organism. Each patient was only included once at the time of the first BSI. Bacteremia was defined as polymicrobial if microorganisms other than *P. aeruginosa *were recovered from the blood culture within a 24 h period. If the bloodstream isolate was a potential skin contaminant (e.g., diphtheroids, *Propionibacterium *spp, *Bacillus *spp, coagulase-negative staphylococci, or micrococci), the presence of an intravascular catheter and the initiation of targeted antimicrobial therapy were required for the diagnosis, as well as at least 1 of the following findings: temperature of >38.0°C or < 36.0°C, chills, and or systolic blood pressure of <90 mmHg. Clinical data were concurrently collected by infection control practitioners using a standardized case report form. The data collected routinely included age, gender, location of the patient (ward vs. ICU), clinical service, duration of hospitalization prior to onset of BSI, predisposing clinical conditions, and bloodstream pathogen. Predisposing clinical conditions were required to be present prior to BSI and included neutropenia (defined as an absolute neutrophil count <500/μl), peritoneal or hemodialysis, and central venous catheters. Sources of secondary BSI were identified by cultures obtained from distant sites that yielded the same pathogen. Adverse outcomes that occurred during the hospital stay were recorded. These included organ failure and mortality (7-day and overall hospital). The clinical condition of each patient was classified daily according to systemic inflammatory response syndrome (SIRS) criteria [SIRS, sepsis, severe sepsis or septic shock] and APACHE II scores from two days prior to the first positive blood culture through 14 days afterwards [8,9]. The severity of underlying disease for each patient was classified using the Charlson weighted comorbidity index [10]. Patients who had nosocomial BSI due to monomicrobial *P. aeruginosa *(MPa) were compared to patients who had nosocomial BSI due to polymicrobial *P. aeruginosa *(PPa) nBSI.

### Definitions

The patient's physiological conditions prior to the BSI and on the day of BSI were assessed using the APACHE II score [9]. The clinical condition of each patient during the bloodstream infection was classified daily as SIRS, sepsis, severe sepsis or septic shock using criteria previously published by the American College of Chest Physicians / Society of Critical Care Medicine (ACCP/SCCM) [8]. Systemic Inflammatory Response Syndrome (SIRS) was defined as two or more of the following: (a) temperature >38°C or <36°C, (b) heart rate >90 beats per minute, (c) respiratory rate >20 breaths per minute or a PaCO_2 _<32 mmHg, or (d) white blood cell count >12 × 10^9^/L or <4 × 10^9^/L or the presence of more than 10% immature neutrophils.

Sepsis was defined as SIRS associated with *P. aeruginosa *isolated from at least one blood culture. Severe sepsis was associated with organ dysfunction, hypotension or systemic manifestations of hypoperfusion. Septic shock was defined as sepsis associated with hypotension unresponsive to intravenous fluid challenge or the need for a vasopressor agent. The presence of organ system failure at the time of BSI and during the clinical course was assessed using the criteria described by Fagon [11]. Nosocomial infection and sources of infection were defined according to Centers for Disease Control and Prevention (CDC) criteria [12]. Adequate empiric antimicrobial treatment was defined as therapy administered within 24 hours after blood culture samples were obtained that included the administration of any antimicrobial agent to which the *P. aeruginosa *and the other co-pathogens were susceptible [13], except when a susceptible aminoglycoside was used in conjunction with another antimicrobial to which the organisms were resistant or when a susceptible aminoglycoside was used alone.

### Microbiological methods

Blood cultures (each consisting of aerobic and anaerobic bottles) were processed at the VCUMC clinical laboratory using the BACTEC^® ^9240 blood culture system (Becton Dickinson, Sparks MD).

### Statistical analysis

For continuous variables, mean values were compared using two sample t-tests for independent samples. Differences in proportions were compared using a Chi-square test or Fisher's exact test when appropriate. Mean values were reported ± 1 SD. All tests of significance are two-tailed. When collinearity was identified, the variable with the greatest measure of association was included in the multivariate analysis. Odds ratios were calculated for all variables. Ninety five percent confidence intervals were calculated for all odd ratios. Variables found to be significant in univariate analysis were then entered into a multivariate model. Alpha was set at 0.05. All statistical analyses were done using the Statistical Package for the Social Sciences software (SPSS, Chicago, IL, USA).

## Results

### Study population and patient characteristics

A total of 160 nosocomial *P. aeruginosa *BSIs were identified at VCUMC during the eight-year study period. Of these, 19 clinically significant episodes of BSI (11.9%) were identified in pediatric patients (<18 years of age), 92 episodes were monomicrobial, and 49 episodes were polymicrobial BSI. Fifteen monomicrobial and 12 polymicrobial BSIs had incomplete medical records. In 16 cases co-pathogens were recovered from the blood culture >24 hours after the isolation of *Pseudomonas aeruginosa*. The remaining 77 monomicrobial and 21 polymicrobial BSIs caused by *P*. *aeruginosa *were included in the analysis.

Proportions and means for the different variables in the two groups are listed in Table 1. There were no significant differences in age or gender between the two groups (p = 0.71 and p = 0.72, respectively). Burn injuries were more commonly seen in cases of polymicrobial *P. aeruginosa *BSI vs. monomicrobial cases (42.9% vs. 10.4%, p = 0.002). Underlying malignancy was more commonly seen in the monomicrobial *P. aeruginosa *BSI cohort (24.7% vs. 4.8%, p = 0.064). No statistically significant differences were observed in the proportion of patients with diabetes mellitus or gastrointestinal complications between the two BSI groups. No difference was also observed in Charlson scores ≥3 between the two groups (19.0% for PPa vs. 29.9% for MPa, p = 0.32). Although the majority of patients acquired *P. aeruginosa *BSI in the intensive care unit setting, no statistically significant differences were noticed between the two comparison groups (90.5% for PPa vs. 81.8% for MPa, p = 0.51). A central venous catheter was present in more than three-quarters of both groups (90.5% in PPa vs. 83.1% in MPa, p = 0.51). Twenty-seven percent of the MPa BSI patients received total parental nutrition compared to 14.3% of PPa BSI patients (p = 0.22). A greater proportion of PPa BSI patients (38.1%) received blood transfusions compared with 26.0% of MPa BSI patients; however, this was not statistically significant (p = 0.28). More than half of patients in both groups needed ventilatory support (71.4% in PPa vs. 61.0% in MPa, p = 0.38) prior to the onset of BSI. There was no difference in the use of any class of antimicrobials prescribed prior to *Pseudomonas *BSI between the two groups (85.7% in PPa vs. 84.4% in MPa, p = 0.88).

**Table 1:** Patient characteristics and outcomes, stratified by polymicrobial infection.

**VARIABLES**	**Polymicrobial (n = 21)**		**Monomicrobial (n = 77)**		**P**
	
	N	%	N	%	
**Demographic characteristics**					
**Age >60 years**	7	33.3	29	37.7	0.71
**Male gender**	17	81.0	46	59.7	0.72
**Mean LOS prior to nBSI (days) ± SD (range)**	26 ± 28.6 (5–121)	-	33 ± 44.7 (2–323)	-	0.38
**Mean hospital stay (days) ± SD (range)**	46.9 ± 32.0 (9–128)	-	69.0 ± 75.4 (5–415)	-	0.05
**ICU stay**	19	90.5	63	81.8	0.51
					
**Underlying conditions**					
**Charlson score ≥3**	4	19.0	23	29.9	0.32
**Burn injury**	9	42.9	8	10.4	0.002
**Diabetes mellitus**	5	23.8	17	22.1	1.0
**Neoplasia**	1	4.8	19	24.7	0.064
**Neutropenia**	0	-	10	13.0	0.11
**Gastrointestinal diseases**	4	19.0	16	20.8	1.0
					
**Therapeutics**					
**Mechanical ventilation**	15	71.4	47	61.0	0.38
**Central venous line**	19	90.5	64	83.1	0.51
**Hemodialysis**	3	14.3	12	15.6	1.0
**TPN**	3	14.3	21	27.3	0.22
**Transfusion**	8	38.1	20	26.0	0.28
**Prior antibiotics**	18	85.7	65	84.4	0.88
					
**Conditions related to the clinical course**					
**APACHE II score ≥20 at BSI onset**	15	71.4	50	64.9	0.58
**Mean time to appropriate antimicrobial therapy (days)**	3.40	-	1.7	-	0.029
**Inadequate antibiotic therapy**	18	85.7	37	48.1	0.002
**Imipenem resistant *P. aeruginosa***	6	28.6	20	26.0	0.81
					
**Outcomes**					
**Respiratory failure**	15	71.4	58	75.3	0.72
**Cardiovascular failure**	7	33.3	30	39.0	0.64
**Renal failure**	11	52.4	28	36.4	0.18
**Hematologic failure**	6	28.6	26	33.8	0.65
**Hepatic failure**	2	9.5	10	13.0	1.0
**7-day mortality**	6	28.6	16	20.8	0.56
**Overall mortality**	8	38.1	37	48.1	0.42

The mean interval between hospitalization and the onset of BSI did not differ between the monomicrobial and polymicrobial groups (26 ± 28.6 days vs. 33 ± 44.7 days, p = 0.38). However, the overall hospital mean length of stay was significantly longer for MPa BSI (69.0 ± 75.4 vs. 46.9 ± 32.0 days, p = 0.05).

### Microbiological features

78.6% of BSIs were caused by monomicrobial *P. aeruginosa *infection (MPa) and 21.4% by polymicrobial *P. aeruginosa *infection (PPa). The most frequent pathogens associated with polymicrobial *P. aeruginosa *BSI were coagulase-negative staphylococci (16.6%), *Acinetobacter baumannii *(16.6%), *Staphylococcus aureus *(12.5%) and *Candida albicans *(12.5%) (Table 2). Polymicrobial infection with more than 2 organisms was seen in 19.1% of PPa BSI cases. No statistically significant differences were observed in the proportion of imipenem resistance in *P. aeruginosa *isolates between the two groups (28.6% for PPa vs. 26.0% for MPa, p = 0.81).

**Table 2:** Characteristics of 26 co-pathogens isolated in 21 cases of polymicrobial *P. aeruginosa *BSI.

**Microorganisms**	**Polymicrobial BSI cases (n = 21)**	
	
	N	%
**Number of agents (associated with *P. aeruginosa*BSI)**		
1	17	80.9
2	3	14.3
3	1	4.8
**Agents (n = 26)**		
CNS	4	15.4
Staphylococcus aureus*	3	11.5
*Enterococcus faecalis*	1	3.8
Enterococcus faecium**	2	7.7
*Streptococcus pneumoniae*	1	3.8
*Acinetobacter baumannii*	4	15.4
*Burkholderia cepacia*	2	7.7
*Enterobacter cloacae*	1	3.8
*Klebsiella pneumoniae*	3	11.5
*Klebsiella oxytoca*	1	3.8
*Serratia marcescens*	1	3.8
*Candida albicans*	3	11.5

CNS = coagulase-negative staphylococci

*Two methicillin-resistant *S. aureus*

**One vancomycin-resistant *E. faecium*

### Clinical course

**Figure 1:**
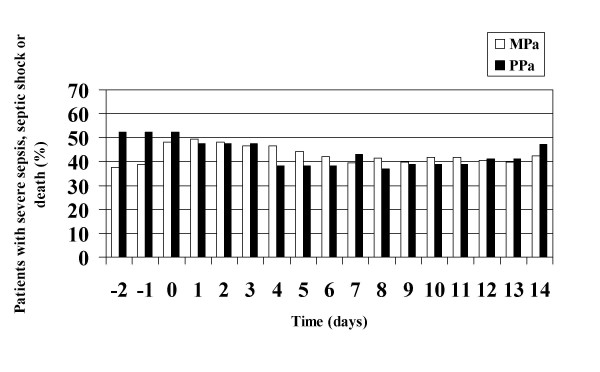
Systemic inflammatory response over time in patients with *P. aeruginosa *nBSI stratified by polymicrobial infection.

**Figure 2:**
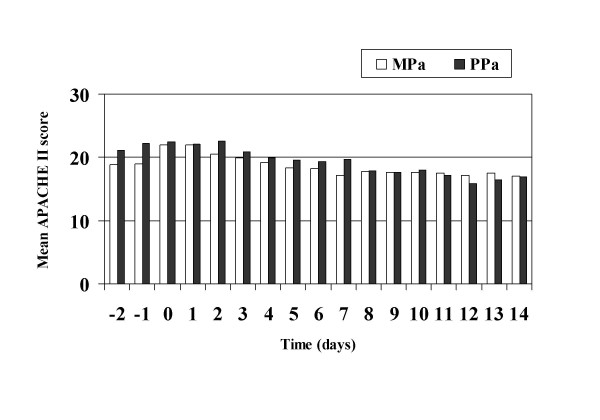
Mean APACHE II scores in patients with *P. aeruginosa *nBSI stratified by polymicrobial infection.

Septic shock occurred in 33.3% of PPa and in 39.0% of MPa (p = 0.64). Progression to septic shock was associated with death more frequently in PPa (OR 38.5, CI95 2.9–508.5) than MPa (OR 4.5, CI95 1.7–12.1). Maximal SIR (severe sepsis, septic shock or death) was seen on day 0 for PPa BSI vs. day 1 for MPa (Figure 1). Median APACHE II scores on the day of BSI were 23 in the PPa group and 22 in the MPa group. There was also no difference in APACHE II scores at 14 days post-BSI diagnosis between the MPa and PPa groups (Figure 2). Appropriate empiric antimicrobials were begun within 24 hours in 59.6% of MPa and in 14.3% of PPa (p = 0.002). In addition the time to adequate therapy was twice as long for patients with PPa infection (3.4 days vs. 1.7 days, p = 0.029). No significant difference was noted in the incidence of organ failure, 7-day mortality, or overall mortality between the two groups as seen in table 1.

Univariate analysis revealed that APACHE II score ≥20 at BSI onset, Charlson score, burn injury, and respiratory, cardiovascular, renal and hematologic failure were associated with death (table 3). Age, malignancy, diabetes mellitus, hepatic failure, gastrointestinal complications, inappropriate empiric antimicrobial therapy, infection with imipenem-resistant *P. aeruginosa*, and polymicrobial infection were not significant predictors of mortality on univariate analysis. Using logistic regression analysis, the following variables were independent predictors for death (table 3): hematologic failure (OR 16.9; CI95 3.9–73.2) and APACHE II score ≥20 at BSI onset (OR 9.7; CI95 1.9–47.9).

**Table 3:** Risk factors for in-hospital mortality in patients with *P. aeruginosa *nosocomial bloodstream infection.

**VARIABLES**	**Died (n = 45)**		**Recovered (n = 53)**		**Univariate analysis**		**Multivariate analysis**	
	
	N	%	N	%	OR	CI 95%	OR	CI 95%
**Age >60 years**	18	40.0	18	34.0	1.3	0.6–2.9		
**Burn injury**	12	26.7	5	9.4	3.5	1.1–10.8	3.2	0.6–18.2
**Diabetes mellitus**	10	22.2	12	22.6	0.9	0.4–2.5		
**Gastrointestinal complication**	6	13.3	14	26.4	0.4	0.1–1.2		
**Neoplasia**	11	24.4	9	17.0	1.6	0.6–4.2		
**APACHE II score ≥20 at BSI onset**	41	91.1	24	45.3	12.7	3.9–39.5	9.7	1.9–47.9
**Charlson score ≥3**	17	37.8	10	18.9	2.6	1.0–6.5	2.7	0.7–10.1
**Respiratory failure**	39	86.7	34	64.2	3.6	1.3–10.1	1.4	0.3–7.2
**Cardiovascular failure**	26	57.8	11	10.8	5.2	2.1–12.7	2.6	0.7–9.3
**Renal failure**	25	55.6	14	26.4	3.5	1.5–8.1	1.3	0.4–4.2
**Hematologic failure**	26	57.8	6	11.3	10.7	3.8–30.2	16.9	3.9–73.2
**Hepatic failure**	8	17.8	4	7.5	2.6	0.7–9.5		
**Inadequate antibiotic therapy**	26	57.8	29	54.7	1.1	0.7–1.7		
**Imipenem resistant *P. aeruginosa***	16	35.6	10	18.9	2.37	0.9–5.9		
**Polymicrobial infection**	8	17.8	13	24.5	0.7	0.2–1.8		

## Discussion

As polymicrobial BSI with *P. aeruginosa *is associated with high mortality, we decided to investigate whether the systemic inflammatory response with polymicrobial *P. aeruginosa *BSI is more intense than the systemic inflammatory response associated with monomicrobial *P. aeruginosa *BSI. During the study period, we found one-fifth of cases were polymicrobial. Since our intention was to analyze the 14-day period after *P. aeruginosa *BSI for the intensity of the systemic inflammatory response, we adopted a more conservative definition of polymicrobial BSI (all organisms isolated in the 24 hour period following the first culture positive for *P. aeruginosa*) rather than the more standard definition (organisms isolated within 48 hours) [14] in order to minimize misclassification of serial monomicrobial infections as polymicrobial infections. It should be noted that the proportion of *Pseudomonas *BSI cases that were polymicrobial was lower at our hospital than in the SCOPE hospitals overall, where 32.7% of the 1,256 nosocomial *Pseudomonas *BSI cases were polymicrobial.

It is also important to note that 19.1% of the patients with PPa BSI had 2 or 3 co-pathogens associated with *P*. *aeruginosa*. Coagulase negative staphylococci (CNS) were found in 15.4% of the PPa BSIs. It is often difficult to determine the pathogenic role of coagulase-negative staphylococci when these organisms are isolated in blood cultures. To avoid this, we utilized more strict criteria for classification of skin flora as pathogens. In addition, in only one of the four polymicrobial BSIs in which coagulase-negative staphylococci (CNS) was a co-pathogen was a third organism also found. However, in this particular case, multiple blood cultures yielded both *Pseudomonas *and CNS, which leads us to believe that both organisms played a pathogenic role.

*Acinetobacter baumannii *was found in 15.4% of the polymicrobial cases. A previous study found that *Acinetobacter *spp. may be a co-pathogen in *P. aeruginosa *polimycrobial BSI [6]. Although both pathogens are non-fermentative Gram-negative bacteria causing nosocomial infections, principally in intensive care units, they show different epidemiologic characteristics [1].

Interestingly, no significant difference was noted between the MPa BSI and PPa BSI cohorts with respect to gastrointestinal complications, such as bowel perforation or gastrointestinal procedures. As such, a gastrointestinal source for the etiology of the polymicrobial BSI is not clearly defined. However, burn injury was more common in PPa BSI (p = 0.002). The loss of the natural cutaneous barrier to infection likely led to microbial colonization and subsequent invasion of the bloodstream [15]. Of note was that malignant conditions were more commonly associated with MPa BSI than PPa BSI.

No difference in APACHE II scores was noted between the two comparison groups during 14 days of follow up. On analysis of severe sepsis, septic shock and death, no statistically significant differences were observed between the MPa and PPa groups. A previous study by Aliaga et al. showed that patients with polymicrobial infection involving *P. aeruginosa *were worse clinically and developed shock more frequently [6]. However, the severity of underlying diseases chosen by these authors was only evaluated by the McCabe classification. In our study, the Charlson weighted comorbidity index and serial APACHE II scores were used to assess the patients' severity of illness (figure 2).

Our study also demonstrated the difficulty in choosing empiric antimicrobial treatment for polymicrobial infections at the time *P. aeruginosa *was isolated. This difficulty is even greater if organisms such as VRE or *Candida *are co-pathogens where it is likely that therapy will be inadequate until all pathogens have been identified [13]. This at least partially explains the difference observed in inadequate empiric treatment in patients with PPa compared to patients with MPa (85.7% vs. 48.1%, p = 0.025). It is also important to note that there was no difference in imipenem resistance between the PPa and MPa groups (p = 0.81).

By univariate analysis several organ dysfunctions (respiratory, cardiovascular, renal and hematologic) were associated with death, but age, hepatic failure, inappropriate antimicrobial therapy, imipenem resistance and polymicrobial infection were not. The clinical belief that polymicrobial BSI portends a worse prognosis than monomicrobial BSI was not demonstrated in our study. Other studies, however, have associated polymicrobial infection with higher mortality [4,5]. Another surprising finding was that the mean length of hospital stay was higher for the monomicrobial group than the polymicrobial group. Thus, our findings suggest that the outcome of *P. aeruginosa *BSI is more closely related to the underlying physiological response to sepsis than it is to polymicrobial infection. However, it must be emphasized that because of the relatively small sample size of our study, a difference of at least 28% between outcome events in the monomicrobial and polymicrobial groups would be necessary to detect a statistically significant difference in SIRS, organ failure or mortality rate. In the SCOPE hospitals overall, the crude mortality of polymicrobial *P. aeruginosa *BSI (42.4%) was significantly higher than that seen with monomicrobial *P. aeruginosa *BSI (34.8%), (p = 0.01). Thus, the smaller sample size in our study could have led to a type II error in our finding of no significant difference in mortality between mono- and polymicrobial BSI.

In conclusion, in patients with *P. aeruginosa *nBSI, one-fifth of cases are polymicrobial, the incidence of septic shock and organ failure is high, patients with PPa BSI are not more severely ill prior to infection than those with MPa BSI, and APACHE II score ≥20 at BSI onset and the development of hematologic failure are independent predictors of death.

## Competing interests

The author(s) declare that they have no competing interests.

## Authors' contributions

ARM participated in the design of the study, collected the data and performed the statistical analysis. GMLB participated in the design of the study and performed the statistical analysis. RPW participated in the design of the study and coordination. MBE conceived of the study, and participated in its design and coordination. All authors read and approved the final manuscript.

## Pre-publication history

The pre-publication history for this paper can be accessed here:


